# Exploring machine learning: a scientometrics approach using bibliometrix and VOSviewer

**DOI:** 10.1007/s42452-022-05027-7

**Published:** 2022-04-11

**Authors:** David Opeoluwa Oyewola, Emmanuel Gbenga Dada

**Affiliations:** 1 Department of Mathematics and Computer Science, Faculty of Science, Federal University of Kashere, P.M.B 0182, Gombe, Nigeria; 2 Department of Mathematical Sciences, Faculty of Science, University of Maiduguri, Maiduguri, Nigeria

**Keywords:** Bibliometrix, VOSviewer, Coupling, Machine learning, Scientometrics

## Abstract

Machine Learning has found application in solving complex problems in different fields of human endeavors such as intelligent gaming, automated transportation, cyborg technology, environmental protection, enhanced health care, innovation in banking and home security, and smart homes. This research is motivated by the need to explore the global structure of machine learning to ascertain the level of bibliographic coupling, collaboration among research institutions, co-authorship network of countries, and sources coupling in publications on machine learning techniques. The Hierarchical Density-Based Spatial Clustering of Applications with Noise (HDBSCAN) was applied to clustering prediction of authors dominance ranking in this paper. Publications related to machine learning were retrieved and extracted from the Dimensions database with no language restrictions. Bibliometrix was employed in computation and visualization to extract bibliographic information and perform a descriptive analysis. VOSviewer (version 1.6.16) tool was used to construct and visualize structure map of source coupling networks of researchers and co-authorship. About 10,814 research papers on machine learning published from 2010 to 2020 were retrieved for the research. Experimental results showed that the highest degree of betweenness centrality was obtained from cluster 3 with 153.86 from the University of California and Harvard University with 24.70. In cluster 1, the national university of Singapore has the highest degree betweenness of 91.72. Also, in cluster 5, the University of Cambridge (52.24) and imperial college London (4.52) having the highest betweenness centrality manifesting that he could control the collaborative relationship and that they possessed and controlled a large number of research resources. Findings revealed that this work has the potential to provide valuable guidance for new perspectives and future research work in the rapidly developing field of machine learning.

## Introduction

Machine learning is a branch of science that studies how systems might be taught to learn on their own and continue to get better with time. In this respect, learning is related to the ability to identify sophisticated patterns and make informed judgments using the data [1]. The sub-discipline of machine learning studies various human learning processes as well as the scientific examination of various learning algorithms and methodologies for a variety of application areas [2]. Machine learning research has prompted researchers and businesses to predict massive mortality incidents [3], uncontaminated water management [4], client segmentation in commercial banking [5], text categorization [6], and crop productivity, like cocoa [7]. Machine learning has also been applied in the field of Optimization [8], intrusion detection [9], email spam filtering [10], image processing [11], crude oil price prediction [12], and others. The problem is that describing the set of all potential decisions given all input combinations is too complicated. To address this challenge, the discipline of machine learning creates algorithms that use strong statistical and computational approaches to uncover knowledge from particular data. In this area, supervised and unsupervised learning approaches are used to solve problems such as classification, prediction, regression, clustering, and association. Machine learning has become a cornerstone of digital technologies and, as a result, a vital aspect of our lives in recent years [13].

The Scientometrics of machine learning is explored in this research, a topic that has increased in prominence in recent years. This paper contributes to knowledge by performing a scientometrics investigation of machine learning publications in several fields of study. A summary of recent progress in ML models development, global structural network, and their application to different fields was presented. Scientometric exploration of several ML types of research that have made meaningful impact among the research community was conducted. Moreover, this paper also investigates the global structure of ML, and also apply Bibliometrix and VOSviewer simulation tools to generate and visualize structure map of 10,814 machine learning research papers published between 2010 and 2020. The Hierarchical Density-Based Spatial Clustering Applications with Noise (HDBSCAN) which is unsupervised learning was used to cluster prediction of authors dominance ranking and its effectiveness was evaluated in this paper.

Scientometrics is a branch of statistics that studies science from a numerical standpoint. The quantification of effect, interpretation of scientific citations, and the creation of indices for use in planning and management contexts are among its main research interests. One of the most extensively utilized scientometric methodologies is citation analysis. It analyzes the frequency, structures, and trajectories of citations in publications using citations in scholarly publications to build relationships to other works or several other scholars. Using citation analysis, scientometrics measures have been developed to evaluate and compare different researchers' research activities based on their productivity. Counting how often journal articles are cited is the most basic of these metrics. They are founded on the notion that notable scholars and publications will be cited more often than others. They are an approach that can be used to objectively describe a researcher's scientific output as a series of numerical data. Scientometrics measures are now routinely used as a key mechanism by numerous funding organizations and promotion panels to examine practically every scientific assessment decision [14].

As a result, scientometrics is becoming a progressively popular subject in the scientific world. Eventually, scientometrics is concerned with not only evaluating the output of research but also with reviewing researcher strategies, socio-organizational frameworks, research and innovation service delivery, the role of science and technology in economic development, policy decisions on research, and technology, and so forth. Researchers all across the globe keep publishing a significant number of scientific publications as knowledge improves. Presently, the volume of data that can be not only saved but also analyzed is growing exponentially. Because of the volume, humans are unable to analyze the data using traditional statistical procedures by hand. Machine learning gives the capabilities for appropriately managing and dealing with massive amounts of data in this context. It also makes it easier to use numerical methods models to make predictions based on experience. It is a significant issue because the work of prediction is regarded as the basis of science.

In this paper, a scientometric analysis of machine learning literature was done. ML algorithms have proven their efficacy in handling large data beyond every reasonable doubt. It is therefore very important to demystify the scientometric of the global structure of machine learning publications. The major contributions of this work include:

i. A survey of recent advances in ML models, global structural network, and their application to classifying, standardizing, and grouping of related publications was presented
ii. Application of scientometric to explore different ML research topics that have attracted the research community
iii. Investigate the global structure of machine learning to determine bibliographic coupling, collaboration among research institutions, co-authorship network of countries, and sources coupling in machine learning; and
iv. Apply Bibliometrix and VOSviewer (version 1.6.16) software tool to create and visualize structure map of source coupling networks of journals, scholars, or different publications using citation, bibliographic coupling, co-citation, or co-authorship relations of 10,814 machine learning research papers published between 2010 and 2020.
v. Apply Hierarchical Density-Based Spatial Clustering of Applications with Noise (HDBSCAN) to clustering prediction of authors dominance ranking.

The rest of this paper is organized as follows: related works are done in Sect. 2. The methodology employed for this work as well as performance measurements is discussed in Sect. 3. The results and the discussion are presented in Sect. 4, and we conclude in Sect. 5.

## Related works

Some research work has used ML techniques to carry out scientometric analysis of related publications where ML was applied to solve some problems. Rincon-Patino, Ramirez-Gonzalez, and Corrales [15] used the SciMAT tool to extracted data of machine learning publications from the Scopus database. Their analysis illustrates the tactical maps of progression and a group of topical networks. The findings give deep insight into machine learning's wide trends. The findings demonstrate that SciMAT is a helpful tool for conducting a scientific mapping study, and they support the notion that ML has a wide range of applications and will remain a fascinating subject of research in years to come. The drawback of their work is that it only covers the period 2007 to 2017. This means that recent publications on the application of ML to various fields were not analyzed. Recently, Klein et al. [16] integrated bibliometric with ML techniques for monoclonal antibody data curation and model development for uncommon illness medication identification. Their approach was used to find novel chemicals that could be used as medication candidates using data gathered from the literature to develop a Bayesian model. The proposed technique was used to evaluate sets of compounds that offer a range of chemically varied structures, and rate these molecules for in vitro testing.

Recently, researchers have focused on the scientometric and bibliometric of the Coronavirus that has ravaged the world. For example, Aristovnik, Raveslj, and Umek [17] conducted a bibliometric examination of COVID-19 publications in the scientific and non-scientific research fields. The study made use of the Scopus database, as well as all relevant and current information on COVID-19 literature, which reached 16,866 in the first six months of 2020. The disadvantage of this research is that several papers on COVID-19 after June 2020 were not examined. Another group of researchers, Haghani et al. [18] carried out a scientometric analysis and exploratory investigation of COVID-19-related papers. The analysis of various recently published COVID-19 literature was not included in this study, which is a limitation. Doanvo et al. [19] used machine learning approaches to analyze the true content of coronavirus publication summaries to find research intersections between COVID-19 and other coronavirus illnesses, as well as research topics that have piqued interest and that require further examination. The downside of this study is that it did not examine various literature on COVID-19 after June 2020.

Furthermore, Dong et al. [20] used topic modeling to understand research flashpoints surrounding COVID-19 and illnesses induced by coronavirus variations. The downside of the study is that it did not examine numerous COVID-19 papers after April 2020. Also, Le et al. [21] used COVID-19 and CORD-19 publishing records to project COVID-19 research activities from the moment the fatal virus was proclaimed a pandemic until May 2020. As a result, research on COVID-19 that was completed after May 2020 was not included in the study. Another work was done by Mao et al. [22] where the authors conducted a global bibliometric and prospective study on the importance and progress of coronavirus research. These authors looked at coronavirus-related literature from 2003 through the second month of 2020. Moreover, Abd-Alrazaq et al. [23] conducted a bibliometric analysis of COVID-19 papers using machine learning. The scientists found 196,630 literature in the CORD-19 database, however, only 28,904 were used in their analysis. The authors, on the other hand, solely utilized ML to divide subjects into topical clusters. The study has one drawback: it only includes COVID-19 publications for a period of seven months (January to July 2020). Several important pieces of the literature were not examined after this period. Furthermore, the study's only result was the percentage of topic and cluster dominance. There is no metric for evaluating the accuracy of the proposed system's machine learning models. In another related work, Colavizza et al. [24] did a scientometric summary of the CORD-19 database. From a scientometric standpoint, the authors looked into the description of publications included in the CORD-19 database. The limitation of the work is that the articles examined are those that are only valid until May 2020. As a result, many COVID-19 studies that were later published were not analyzed.

Abualigah et al. [48] developed the Arithmetic Optimization Algorithm (AOA). It is a novel meta-heuristic technique that takes advantage of the distribution behavior of the major arithmetic operators in mathematics. AOA is scientifically developed and deployed to optimize processes across a wide range of search spaces. To demonstrate AOA's universality, its performance is tested on twenty-nine benchmark functions and various real-world engineering design issues. Different situations were used to assess the proposed AOA's performance, convergence tendencies, and computing complexities. Simulation results showed that AOA performed better than the other eleven popular optimization algorithms compared in the paper. Experimental results indicated that AOA gives highly promising outcomes in handling hard optimization issues. In terms of solution quality and computational performance, AOA outperforms other famous optimization techniques for the majority of the problems studied. Furthermore, AOAs’ results demonstrated their supremacy in evading being stuck in the local optima.

Abualigah et al. [49] present a novel multilevel thresholding method centered on the Evolutionary Arithmetic Optimization Algorithm (AOA). The algorithm was instigated by the arithmetic operators used in science. The proposed strategy, DAOA, uses the Differential Evolution method to improve AOA local research. Employing Kapur's measure between-group variance functions, the presented approach is applied to the multilevel thresholding problem. The proposed DAOA is used to analyze images, which are comprised of eight standardized test images from two different classes: nature and CT COVID-19 images. The effectiveness of the developed DAOA method was evaluated against existing multilevel thresholding techniques. The findings indicated that the DAOA process is better than other similar methods and generates enhanced results.

In summary, the research gaps identified from this literature showed that machine learning has been applied to solve problems in different fields of human endeavor. Moreover, the bulk of the COVID-19-related articles examined in the studies have narrow dates, around three months following the commencement of the COVID-19 pandemic. As a result, some subsequent papers were not examined. Furthermore, rather than focusing on COVID-19, numerous investigations looked into the literature related to a variety of coronaviruses. As a result, the findings of COVID-19-related research were integrated with those connected with other coronavirus variations. Furthermore, a small number of COVID-19-related papers were included in numerous studies. Furthermore, many research did not look into the subject that previous studies had looked into, instead of focusing on the metadata of those studies (such as countries, author name, author affiliation, total citations, bibliometric items, source journals, and others). Finally, rather than employing machine learning approaches, the classification of subjects across different studies was done manually. This paper will conduct a wide scientometric analysis of existing machine learning publications that have been applied to different fields to adequately address the identified gaps in the literature.

## Methods

### Data collection

Dimensions is a comprehensive worldwide academic database that includes more than 1.4 billion citations, data sets, patents, and policy papers from several millions of research publications [25]. The Dimensions database includes several features which may be utilized for bibliometric study, including the title, author, institution, country, year of publication, grants, clinical trials, and keywords [26, 27]. As a dependable data source for scientific analysis with applications in mathematical research, the Dimensions database has received greater attention recently [28–31]. We carried out a search in Dimensions database for articles using machine learning from 2010 to 2020. All documents were incorporated and no constraint of language has been established. By carefully examining the obtained papers, we confirmed the reliability of our search approach. The information retrieved from the Dimensions database are Publication ID, DOI, Title, Abstract, Source title, PubYear, Volume, Issue, Pagination, Authors, Authors affiliations, Dimensions URL, Times cited, and Cited references. All information was collected and stored in CSV format from the retrieved Dimensions Database.

### Visualization and scientometrics analysis

We focused on using scientometric analysis to detect annual scientific production, co-citation network of the authors, collaboration network, documents coupling, research frontiers, and other scientometric information in machine learning. The scientometric analysis comprises the construction and graphical display of bibliometric maps [32]. In this study, we employed bibliometric analysis tools on the Dimensions data. Bibliometrix [33] has been used for bibliographical information extraction, analysis, and visualization such as authors co-citation network, institutions collaboration networks. Massimo Aria and Cuccurullo developed Bibliometrix. This is an open-source research tool for scientific and bibliometric quantitative research, which includes all major bibliometry testing methodologies [34]. VOSviewer (version 1.6.16; Leiden University) [35] has been used for collecting bibliographic data on collaborative networks, documents, and sources of researchers, authors and countries. VOSviewer is a software tool that creates maps using network data to build networks of scientific articles, scientific journals, scientists, research organizations, countries, and keywords. VOSviewer creates network-based maps, visualizes and explores maps. VOSviewer supports three map visualizations: the visualization of the network, the overlay visualization, and the visualization of density [36].

### Hierarchical density-based spatial clustering of applications with noise (HDBSCAN)

Hierarchical Density-Based Spatial Clustering of Applications with Noise (HDBSCAN) is unsupervised learning. HDBSCAN is a hierarchical clustering algorithm that improves on Density-Based Spatial Clustering of Applications with Noise (DBSCAN) by using a strategy to extract a flat clustering based on cluster stability [37]. HDBSCAN mathematical representation is as follows:

1 $${\text{max}}\left\{ {d\left( {x_{r} } \right), d\left( {x_{n} } \right),d\left( {x_{r} ,x_{n} } \right)} \right\}$$

2 $$S\left( {c_{i} } \right) = \mathop \sum \limits_{{x_{j} \in c_{i} }} \left( {\alpha_{\max } \left( {x_{j} ,c_{i} } \right) - \alpha_{\min } \left( {c_{i} } \right)} \right) = \mathop \sum \limits_{{x_{j} \in c_{i} }} \left( {\frac{1}{{\delta_{\min } \left( {x_{j} ,c_{i} } \right)}} - \frac{1}{{\delta_{\max } \left( {c_{i} } \right)}}} \right)$$

The optimization problem for the sum of cluster stabilities is given as:

3 $$\mathop {\max }\limits_{{\beta_{2} , \ldots ,\beta_{k} }} Q = \mathop \sum \limits_{i = 2}^{k} \beta_{i} S\left( {c_{i} } \right)$$

4 $$s.t \beta_{i} \in \left\{ {0,1} \right\}, i = 2, \ldots ,k$$

$$\mathop \sum \limits_{{Q \in I_{h} }} \beta_{j} = 1, \forall h \in L$$

where $d\left( {x_{r} ,x_{n} } \right)$ is the normal distance, $d$ is the core distance, $S\left( {c_{i} } \right)$ is the stability, $\alpha$ is the density value, $c_{i}$ is the cluster, $\beta_{i}$ is the Boolean indicator, $L$ is the leaf cluster, $I_{h}$ is the set of clusters on the paths from leaves to the excluded root.

### Clustering predictions of authors dominance ranking

This study proposed clustering authors dominance ranking by extracting the author’s name, dominance factor, number of authored articles, number of single-authored articles, number of multi-authored articles, number of first-authored articles, author ranking by number of articles, and author ranking by dominance factor from the dominance function equation. A total of 5421 of the author dominance ranking during 2010–2021 was extracted. Cluster analysis is a strong data mining method for identifying distinct groups of authors and other sorts of behaviors that are not identified by dominance ranking. It aids in the discovery of groups in unlabeled data, with components belonging to the same group sharing comparable dataset feature values. While clustering has a wide range of applications, we will be focusing on clustering for exploratory data analysis. Exploratory data analysis refers to the act of looking for intriguing patterns in a data collection, such as author dominance ranking, to develop new hypotheses or research questions regarding the data set. In this section, Hierarchical Density-Based Spatial Clustering of Applications with Noise (HDBSCAN) will be used to forecast clustering of author dominance rankings. HDBSCAN is a density-based clustering method that builds a cluster hierarchy tree and then extracts flat clusters from it using a specified stability metric. HDBSCAN builds a hierarchy for all potential epsilon values concerning minimum cluster size, rather than selecting clusters based on a global epsilon threshold.

## Results

**Fig. 1:**
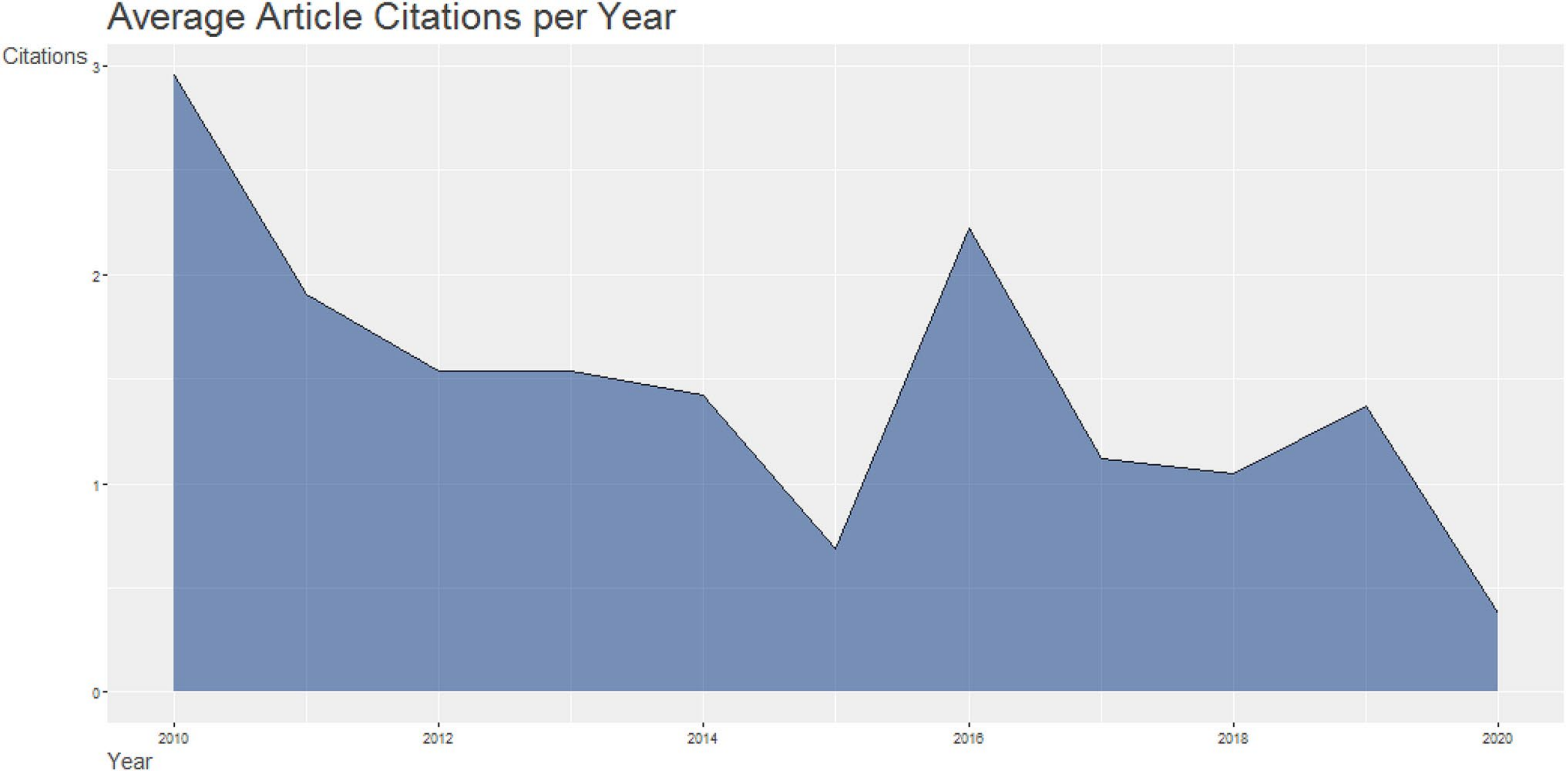
Average article citations per year

**Fig. 2:**
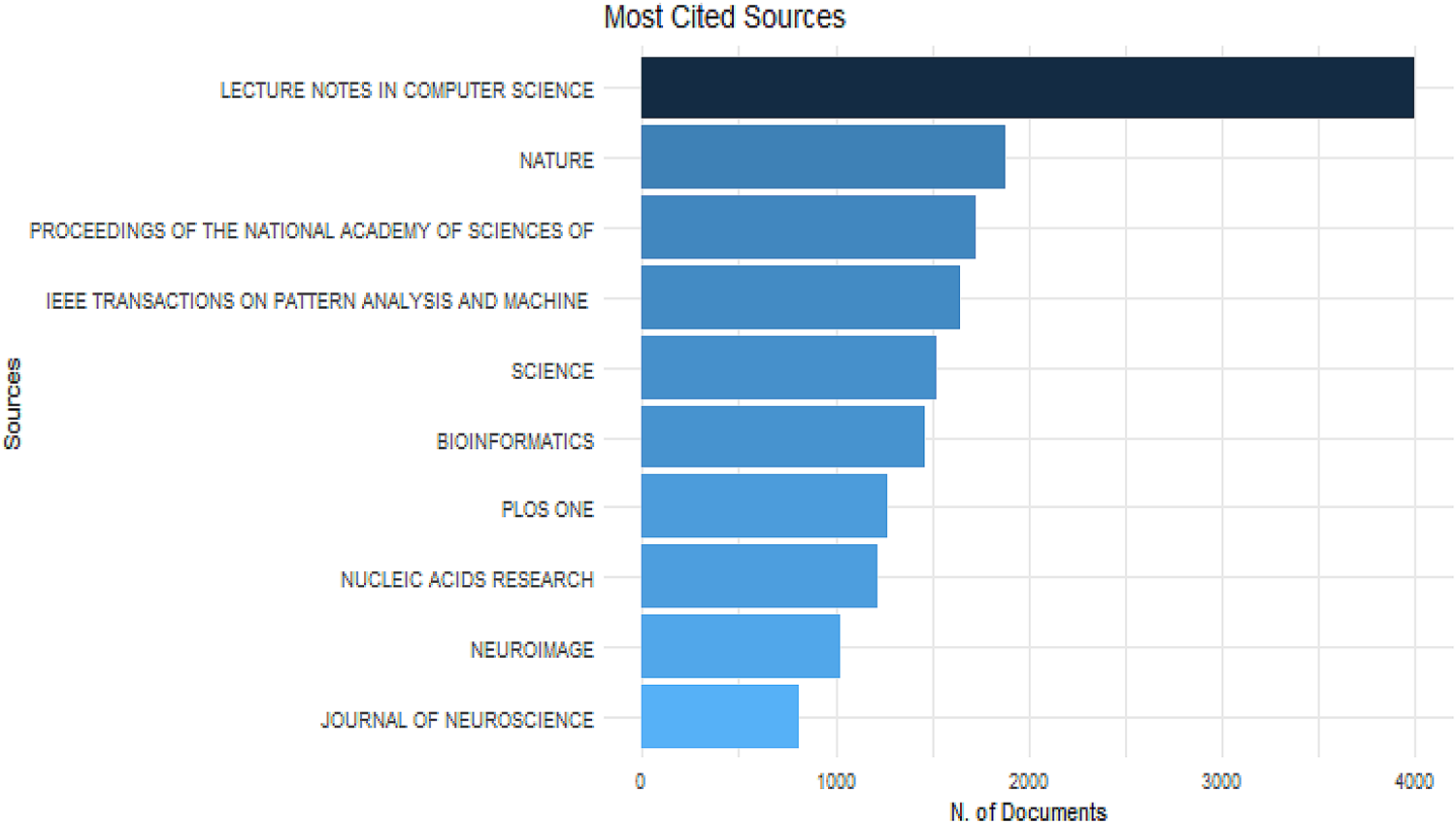
Most cited sources

The data set was extracted from the Dimensions bibliographic database. It includes all publications of the document types such as article, letter, proceedings paper, and so on, published between 2010 and 2020. The number of documents in the data set is 10,814 while the number of references is 161394. The single-authored documents have 2926 while the multi-authored documents are 20,788. This shows that authors prefer multi-authored documents in machine learning to be published as single-authored. The statistics for the data set are summarized in Table 1. Figure 1 displays the average article citations per year of both single and multi-authored. The year 2010 has the most cited document followed by the year 2016. 2020 has the least cited documents. The most cited Source is Lecture Notes in Computer Science with 3999 articles followed by Nature journal with 1879 articles. The least was from Neuroimage and Journal of NeuroScience with 1020 and 809, respectively, as shown in Fig. 2.

**Table 1:** Main Information about the data

Description	Results
TimeSpan	2010–2020
Sources (journal, books, etc.)	4462
Documents	10,814
References	161,394
Authors	23,714
Authors of single-authored documents	2926
Authors of multi-authored documents	20,788

**Fig. 3:**
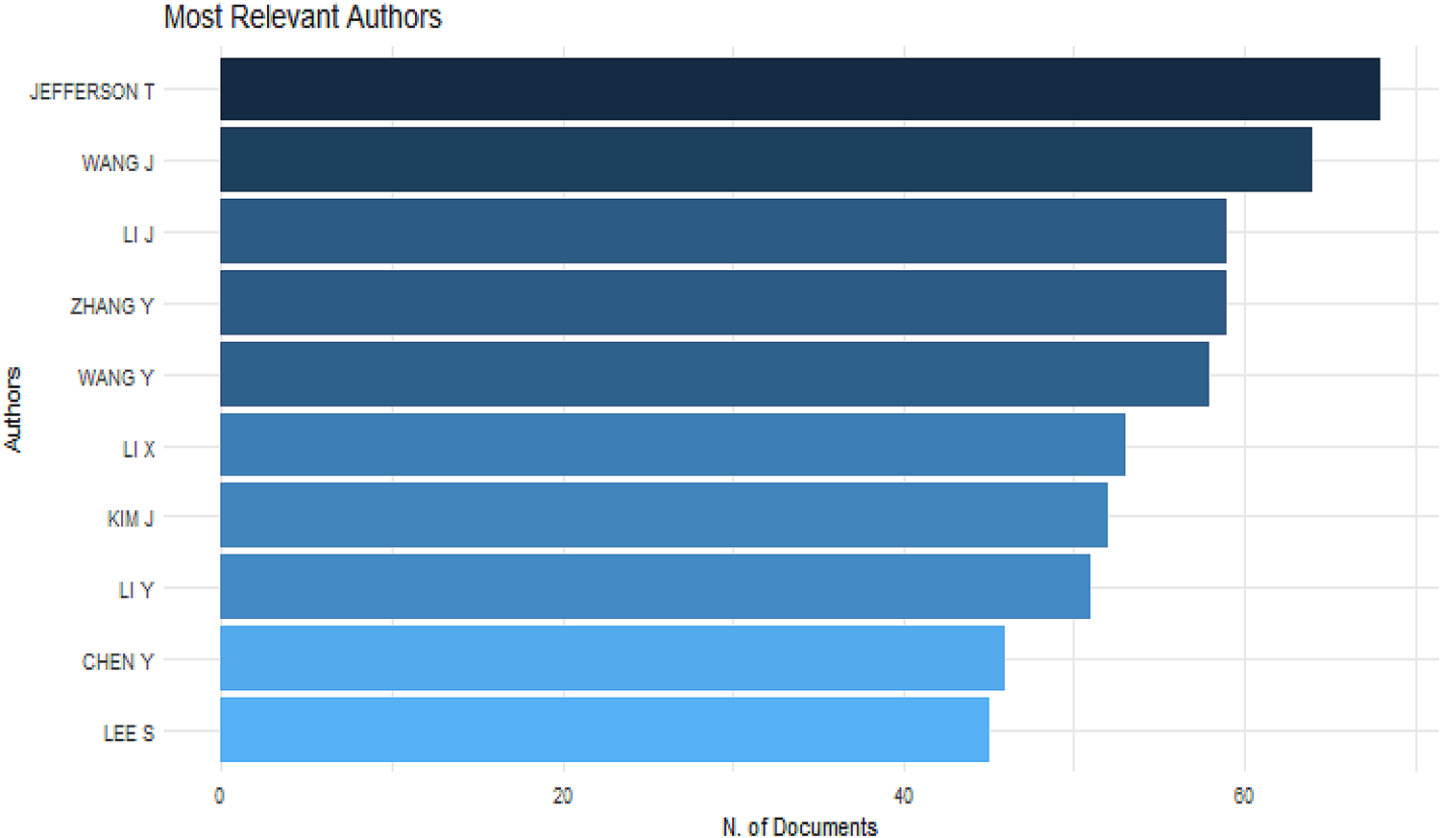
Most relevant authors in machine learning

**Fig. 4:**
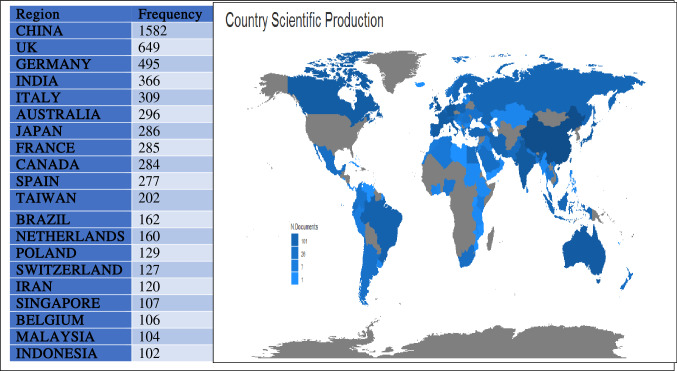
Country scientific production

A measure of the frequency with which the average article is cited for a certain year in a journal can be defined as a source impact factor. It is used to assess a journal's importance or rank by counting the number of times articles are cited. In this study, we use three different measures that are frequently used to measure the impact factors of the journal such as h_index, g_index, and m_index. PLOS ONE ranked high with an h_index of 28 while IEEE TRANSACTIONS ON IMAGE PROCESSING and SENSORS ranked high with 33 g_index if we considered using g_index (see Table 2). However, PLOS ONE ranked first using m_index with a 2.3 impact factor. This shows that articles published in PLOS ONE have been cited more than other journals. Figure 3 presents the most relevant top 10 authors in machine learning research. Jefferson T. (68 publications) ranked first among all authors, followed by Wang J (64 publications), Li J and Zhang Y (59 publications), and Wang Y (58 publications). Figure 4 is the scientific production of machine learning from several countries, in terms of the paper published. The geographic distribution of papers based on all authors' affiliations is concentrated in Asia countries with China (1582 publications), ranked first among all the countries, followed by European countries (UK (649 publications), Germany (495 publications)).

**Table 2:** Source impact factor

Source	h_index	g_index	m_index	TC	PY
Lecture notes in computer science	18	27	2.25	1769	2014
Advances in intelligent systems and computing	8	11	1	319	2014
PLOS one	28	15	2.3	3116	2010
Proceedings of Spie	6	7	0.5	136	2010
IEEE access	11	25	1.2	699	2013
Communications in computer and information science	5	5	0.71	92	2015
International journal of engineering and advanced technology	1	1	0.3	6	2019
Sensors	15	33	1.25	1130	2010
Journal of physics conference series	4	10	0.36	120	2011
BIORXIV	8	10	1	150	2014
BMC bioinformatics	14	27	1.17	752	2010
Studies in computational intelligence	11	18	1.375	400	2014
Contemporary sociology a journal of reviews	2	2	0.2	10	2012
Neural computing and applications	14	32	1.67	1095	2010
International statistical review	3	6	0.25	39	34
Lecture notes of the institute for computer sciences, social informatics and telecommunications engineering	5	8	1	95	2017
IEEE transactions on image processing	17	33	1.42	2557	2010
Multimedia tools and applications	10	14	0.83	267	2010

**Fig. 5:**
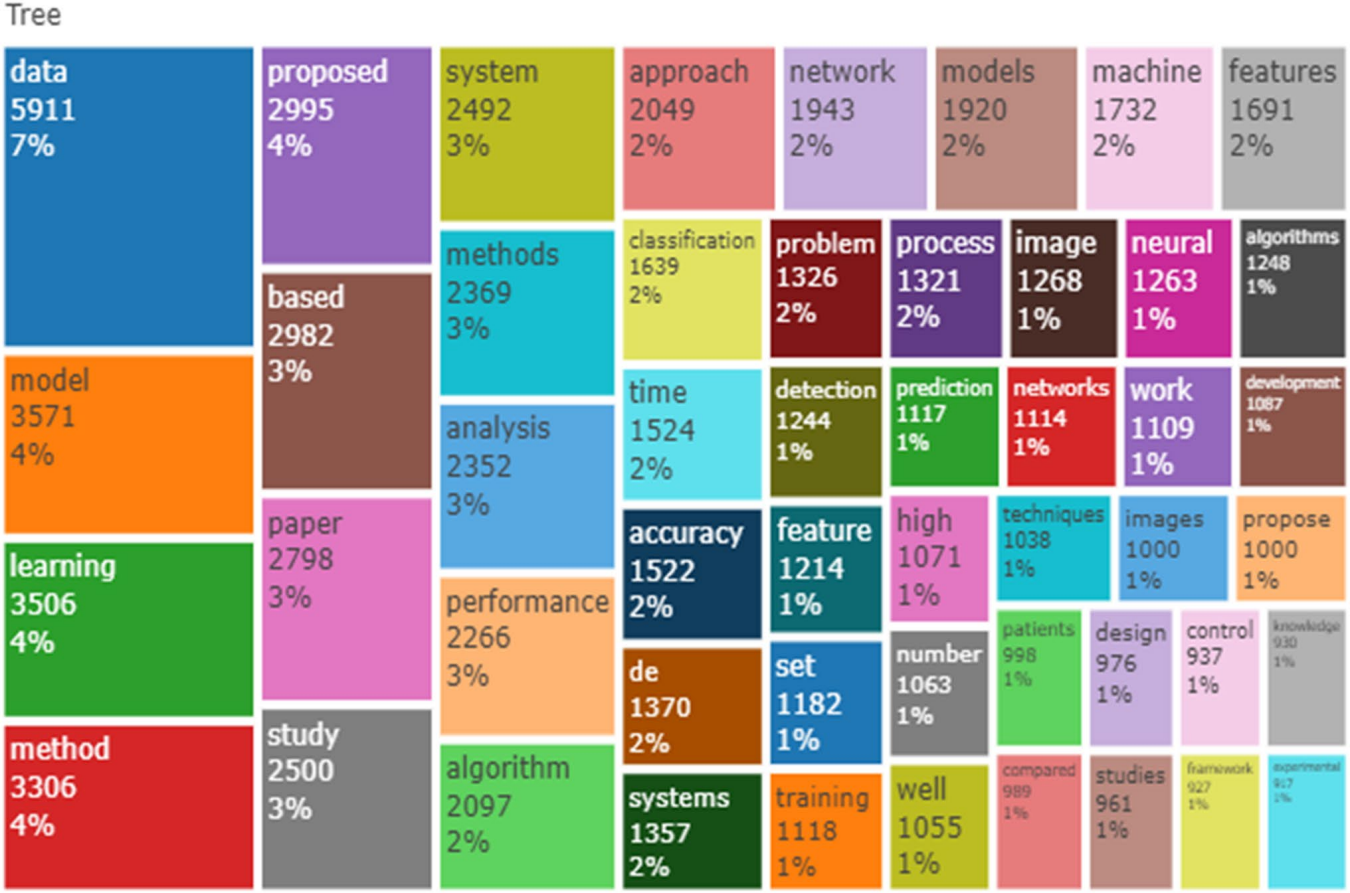
Abstracts TreeMap of machine learning

Table 3 displays the ten most globally cited documents. It contains four columns: Paper, Doi, Total Citation (TC), Normalized Total Citation (NTC), and Country. The author Oostenveld R in computational intelligence and neuroscience journal ranked first with 4894 total citations followed by Babanko B of IEEE transactions on pattern analysis and machine intelligence journal with 1553 total citations. Four out of ten of the global cited documents are concentrated in IEEE journal while seven out of ten of the global cited country is concentrated in the United States of American. Figure 5 is the frequency word of the abstract of the machine learning shown on a TreeMap. DATA were the most frequently used word in the abstract with 5911 (7%) occurrence, while MODEL was the most frequent word with 3571 (4%). This shows the importance of data and modeling in machine learning.

**Table 3:** Most global cited documents

Paper	DOI	TC	NTC	Country
Oostenveld, 2010, computational intelligence and neuroscience [38]	10.1155/2011/156869	4894	150.64	Netherlands
Babenko, 2010, IEEE transactions on pattern analysis and machine intelligence [39]	10.1109/TPAMI.2010.226	1553	47.80	United States
Cai, 2010, IEEE transactions on pattern analysis and machine intelligence [40]	10.1109/TPAMI.2010.231	1294	39.83	United States
Barnich, 2010, IEEE Transactions On Image Processing [41]	10.1109/TIP.2010.2101613	1201	36.97	Belgium
Goferman, 2011, IEEE transactions on pattern analysis and machine intelligence [42]	10.1109/TPAMI.2011.272	1150	60.43	Israel
Graveley, 2010, nature [43]	10.1038/NATURE09715	1104	33.98	United States
Reich, 2010, nature [44]	10.1038/NATURE09710	1103	33.95	United States
Roy, 2010, science [45]	10.1126/SCIENCE.1198374	932	28.69	United States
Cao, 2010, journal of operations management [46]	10.1016/J.JOM.2010.12.008	837	25.76	United States
Shulaev, 2010, nature genetics [47]	10.1038/NG.740	834	25.67	United States

**Fig. 6:**
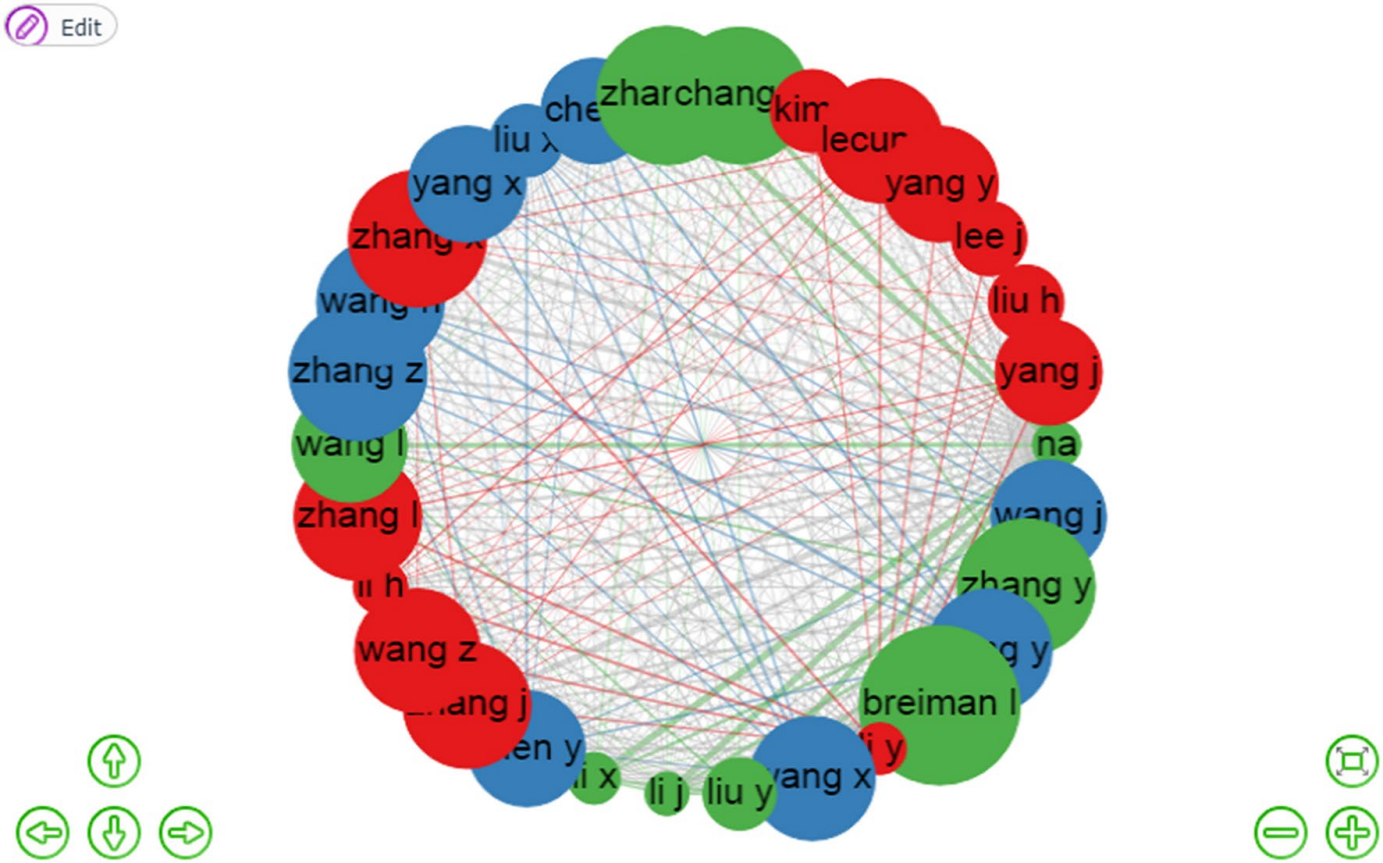
Authors co-citation network in machine learning

Bibliometrix can be used to acquire an overview of the most often mentioned publications, the citation relationships between these publications, the time order of publications, and the assignment of publications to clusters for a certain number of publications. In this study, we want to better comprehend the co-citation published by authors from various levels of clusters. Figure 6 shows a visualization of bibliometrix in three groups of 30 of the most often cited papers. Each publication of the author is shown in a circle and is denoted by the author's name. The color of a publication shows the cluster to which the author's publication belongs, with red, blue, and green corresponding to clusters 1, 2, and 3, respectively. We examined the co-citation patterns of 30 productive authors and created a co-citation map. We discovered that few authors tended to collaborate with a large group, resulting in three primary author clusters, each with one or two main authors. According to the social network analysis, it proved that the research co-citation network in machine learning is very strong. The component analysis found that three research groups can be regarded as the backbone in this field. Therefore, researchers in machine learning should strengthen their co-citation network to improve the development and academic level of this field. Each node of the figure represents an author, and the connections among the nodes represent the co-citation relationships among authors. The weight of a link indicates the number of publications co-authored by two scholars. In this author’s co-citation network, the highest betweenness of wang j, wang x, and yang x was within the range of 4–6.4, indicating that they played a pivotal role in the co-citation network in cluster 2(blue). In cluster 1 (red), zhang x, li x, and liu y obtained the highest betweenness centrality manifesting that they could control co-citation relationship and that he possessed and controlled a large number of research resources. However, in cluster 3(green), li y, yang j, and wang z obtained the highest betweenness within the range of 1–7. In a co-citation network, the closer the distance between one author and the other, the easier it is to exchange information and build a cooperative research relationship (Appendix for Table 7).

**Fig. 7:**
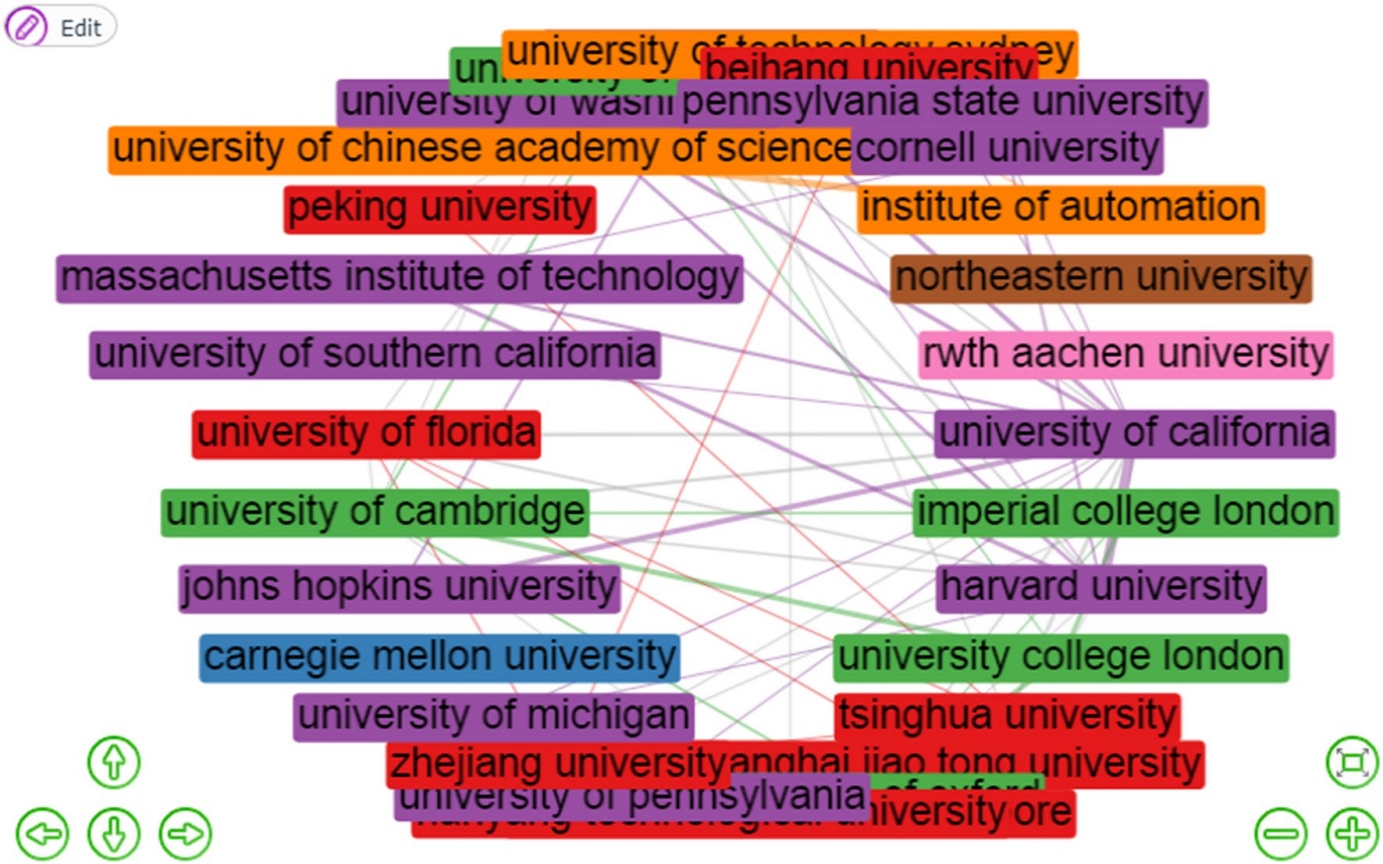
Structure map of institutions collaboration network of machine learning

Figure 7 is the structure map of the institution collaboration network of machine learning. There are 11 clusters of institution collaborative networks structure but only three have the highest number of institutions and these are clusters 1, 2, and 3. Each node of the figure represents an institution, and the connections among the nodes represent the collaborative relationships among institutions. The weight of a link indicates the number of publications co-authored by two scholars in different institutions. In this collaboration network, the highest degree of betweenness centrality was obtained from cluster 3 with 153.86 from the University of California and Harvard University with 24.70. In cluster 1, the national university of Singapore has the highest degree betweenness of 91.72. Also, in cluster 5, the University of Cambridge (52.24) and imperial college London (4.52) having the highest betweenness centrality manifesting that he could control collaborative relationships and that they possessed and controlled a large number of research resources. Furthermore, it was observed that there is no strong level of cooperation among other institutions (Appendix Table 8).

**Fig. 8:**
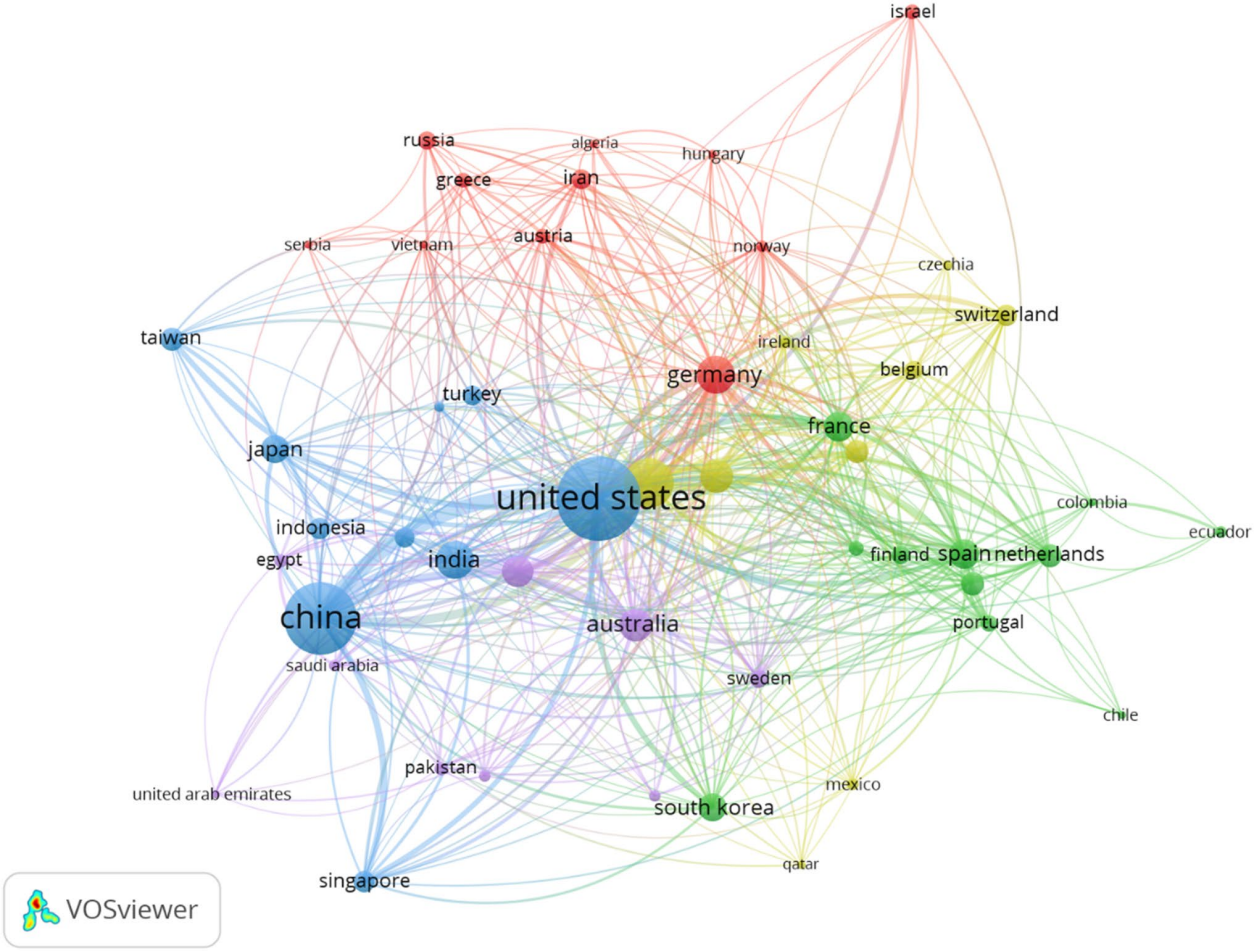
Structure map of countries co-authorships in machine learning

**Fig. 9:**
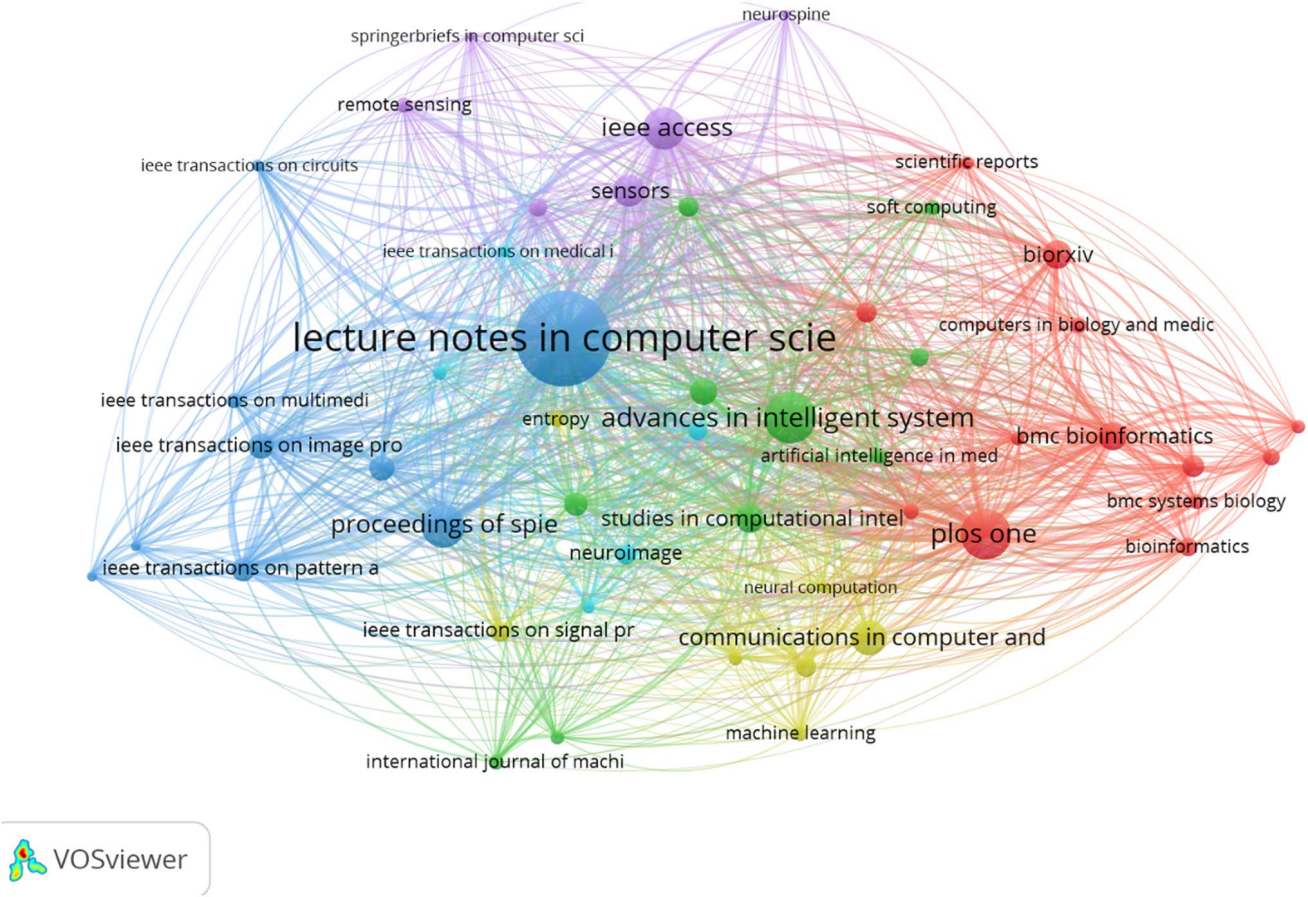
Structure map of sources coupling in machine learning

Figure 8 provides a VOSviewer (version 1.6.16) of visualization of the 50 most co-authorship countries in five clusters. The minimum number of documents of a country is set as 5 while the minimum number of citations of a country is set as 2. As shown in Fig. 8, the co-authorship analysis of countries reflects the collaboration relationship between countries in this field, as well as the degree of collaboration. The larger nodes represent the most productive countries in the field of machine learning; the thickness and length of links between nodes represent the cooperative relationship between countries. Figure 8 shows the 50 most productive countries in the field of machine learning from 5 collaboration clusters, which were distinguished by different colors. The countries with the highest total link strength were the USA with 1352 documents and the link strength of 601 followed by China with 413 total link strength and 1014 documents. The United Kingdom was in the third position with 470 documents and total link strengths of 388 (see Appendix Table 9). As shown in Fig. 9, we used VOSviewer to build a visualization structural network map of the top 50 sources (journals books, etc.) in 5 clusters. The minimum number of documents of a source is set at 5, while the minimum number of citations of a source is set at 2. The larger node represents the most productive source in the field of machine learning. Lecture notes in computer science have the highest total link length of 8618 and 467 documents followed by IEEE access with 94 documents and total link strength of 3500. The third position is PLOS ONE with total link strength of 1893 and 129 documents (see Appendix Table 10).

**Fig. 10:**
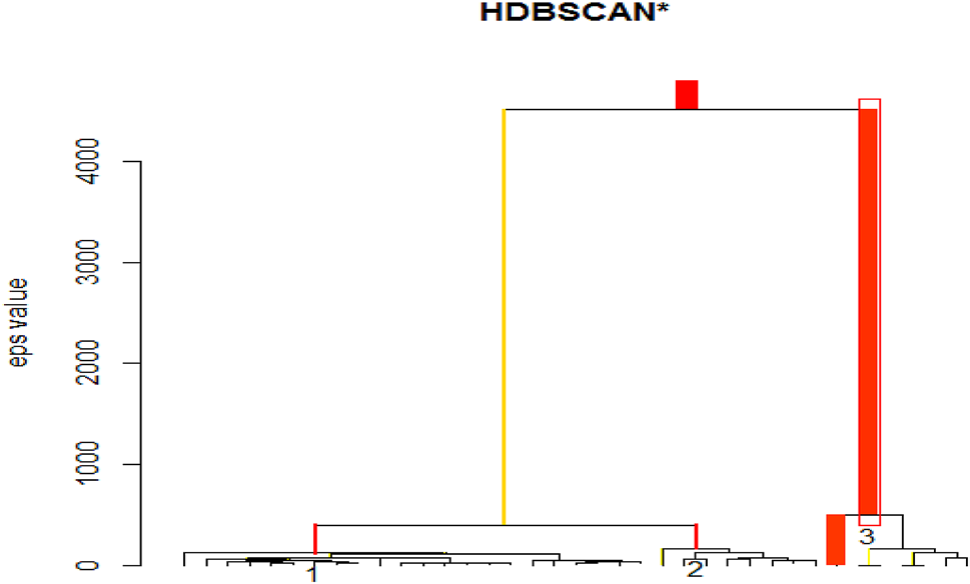
Hierarchical Density-Based Spatial Clustering of Applications with Noise (HDBSCAN) of Authors Dominance Ranking Tree

Table 4 provides the authorship patterns of publication in machine learning of this table where the majority of the articles were contributed from the Multi authors with 9994(60.17%). The second position of the articles was contributed by the first authors with 6423(38.67%). The third position of the articles was contributed by the single authors and five authors were contributed 191(1.15%). This shows that researchers in machine learning published more as Multi-authored than single-authored. Figure 10 is the Hierarchical Density-Based Spatial Clustering of Applications with Noise (HDBSCAN) of Authors Dominance Ranking Tree. HDBSCAN cluster authors dominance ranking into three and these are cluster 1, 2, and 3. In this clustering prediction of authors dominance ranking, the highest degree was obtained from cluster 3 with 8.18 followed by cluster 1 with 2.54 as shown in Table 5. Table 6 consists of the 10 most ranking authors from each cluster. In cluster 3, Bennett RJ, Joughin L, and Sachnev V were having a membership probability of 1 and total articles of 5 while Berlin I was having the highest dominance ranking with total articles of 14 but a membership probability of 0. This shows that the author Berlin I is having a stability problem while Bernett RJ, Joughim L, and Sachnev V are very stable with other researchers. In cluster 2, Dai Y, Dehzangi O, and Feng Y are more stable with other researchers than Gao H and Huang K with 0 membership probability. In cluster 1, most of the authors are having higher total articles but lower membership probability. This shows that authors with higher total articles are not stable.

**Table 4:** Authorship pattern of publication in machine learning

Authors	Article	Cumulative	% Article	% Cumulative
First	6423	6423	38.67	38.67
Single	191	6614	1.15	39.82
Multi	9994	16,608	60.17	100.00

**Table 5:** Cluster scores of authors dominance ranking

Cluster 1	Cluster 2	Cluster 3
2.5422	1.6105	8.1829

**Table 6:** Authors dominance ranking stability

S/No	Author	Total articles	Cluster	Membership_prob
1	Berlin I	14	3	0
2	Gray A	13	3	0.08
3	Bowen WG	6	3	0.5806
4	Gaggioli A	6	3	0.5806
5	Jarvis S	6	3	0.58003
6	Moser M	6	3	0.5806
7	Bennett RJ	5	3	1
8	Innis H	5	3	0.94306
9	Joughin L	5	3	1
10	Sachnev V	5	3	1
11	Lu Z	6	2	0.13506
12	Gao H	5	2	0
13	Huang K	8	2	0.20189
14	Dai Y	4	2	1
15	Dehzangi O	4	2	1
16	Feng Y	4	2	1
17	Han W	4	2	1
18	Hu H	4	2	1
19	Ma J	4	2	1
20	Nguyen N	4	2	1
21	Luo Y	9	1	0.52198
22	Chen S	15	1	0.66437
23	Yang D	8	1	0.67509
24	He Y	7	1	0.68969
25	Kim W	7	1	0.68969
26	Ma H	7	1	0.68969
27	Yuan Y	7	1	0.68969
28	Wang T	13	1	0.62296
29	Chen K	12	1	0.66437
30	Zhao Z	12	1	0.66437

## Conclusion

Through the aid of scientometric quantitative analysis and visualization network map of the data extracted from the Dimensions database, the current study reveals the average article cited per year, most cited sources, most relevant authors, countries scientific production, most global cited documents, authors co-citation, institutions collaboration, countries co-authorships in machine learning research. Application of Hierarchical Density-Based Spatial Clustering of Applications with Noise (HDBSCAN) to clustering prediction of authors dominance ranking was implemented. The result shows that most of the authors that are having higher total articles are having a lower membership probability. This shows that authors with higher total articles are not stable with other researchers. We anticipate that by completely describing the trends in machine learning research, our findings will provide useful insight into future research paths and perspectives in this rapidly evolving subject. Many avenues for significant future work exist. Future work should explore several unsupervised learning on author dominance ranking to obtain the prediction of the cluster. Although this study concentrated on the Dimensions database, other databases can be applied such as Scopus, Web of Science, Cochrane Library, and Pubmed.

## Appendix

See Tables

**Table 7:** Authors co-citation network

Node	Cluster	Betweenness	Closeness	Page rank
Na	1	39.862	0.034483	0.100977
Zhang y	1	6.778026	0.034483	0.052232
Breiman l	1	0.449603	0.025	0.024264
Liu y	1	2.882957	0.03125	0.03954
Li j	1	1.73319	0.030303	0.035369
Li x	1	2.930381	0.033333	0.038452
Zhang j	1	2.347137	0.03125	0.035592
Li h	1	0.642986	0.027778	0.028383
Wang l	1	0.641168	0.027027	0.026202
Zhang x	1	1.359754	0.030303	0.028648
Chang c	1	0.284345	0.02381	0.019648
Lecun y	1	0.525548	0.025641	0.022815
Wang j	2	4.945939	0.033333	0.046948
Wang y	2	4.680509	0.032258	0.050481
Wang x	2	4.43975	0.032258	0.04237
Chen y	2	1.791622	0.03125	0.033844
Zhang z	2	1.01673	0.029412	0.029029
Wang h	2	1.38146	0.027778	0.031242
Yang x	2	0.15204	0.02381	0.020418
Liu x	2	1.358801	0.028571	0.028303
Chen j	2	0.266828	0.025	0.02309
Zhang h	2	0.323104	0.025641	0.023837
Liu h	2	0.777602	0.028571	0.026198
Li y	3	7.690078	0.034483	0.05035
Wang z	3	1.45417	0.027778	0.027624
Zhang l	3	1.33107	0.029412	0.030797
Kim j	3	0.265566	0.021739	0.015888
Yang y	3	0.611621	0.026316	0.022786
Lee j	3	0.462732	0.022222	0.017127
Yang j	3	1.613288	0.028571	0.027548

^7^,

**Table 8:** Institutions collaboration networks

Node	Cluster	Betweenness	Closeness	PageRank
National university of Singapore	1	91.7238	0.0069	0.04302
Nanyang technological university	1	0	0.00588	0.01461
Shanghai Jiao tong university	2	0	0.00629	0.01545
Zhejiang university	2	50.3483	0.00725	0.04838
Beihang university	2	0	0.00613	0.01095
University of California	3	153.865	0.00758	0.15613
Harvard university	3	24.7061	0.00719	0.09291
University of Pennsylvania	3	0	0.00637	0.00968
University of Michigan	3	0	0.00645	0.01363
Johns Hopkins university	3	0	0.00641	0.02469
University of southern California	3	0	0.00637	0.00968
Massachusetts institute of technology	3	0	0.00649	0.02863
University of Washington	3	0	0.00671	0.02787
University of Toronto	3	12.1296	0.00699	0.04273
STANFORD university	3	1.50044	0.00685	0.03614
Pennsylvania state university	3	0	0.00637	0.00968
Cornell university	3	0	0.00649	0.02002
Tsinghua university	4	34.3056	0.00714	0.04059
University of Florida	4	14.8776	0.00704	0.04504
Peking university	4	0.18571	0.00629	0.01467
Imperial college London	5	4.51594	0.0069	0.03503
University college London	5	1.404	0.00662	0.04638
University of oxford	5	1.19615	0.0068	0.0358
University of Cambridge	5	52.2418	0.0073	0.06542
Carnegie Mellon university	6	0	0.00115	0.00546
University of Chinese academy of sciences	7	0	0.00467	0.03467
University of technology Sydney	8	48	0.00602	0.01886
Institute of automation	9	25	0.00529	0.04295
Northeastern university	10	0	0.00115	0.00546
Rwth Aachen university	11	0	0.00115	0.00546

^8^,

**Table 9:** Co-authorship structural network in machine learning

Country	Documents	Total link strength
United States	1352	601
China	1014	413
United Kingdom	470	388
Germany	290	234
Australia	215	205
France	171	161
Canada	195	158
Spain	171	161
Italy	216	123
Netherlands	108	104
Singapore	87	88
Switzerland	91	88
India	272	65
South Korea	148	65
Sweden	73	61
Japan	154	56
Belgium	65	53
Denmark	49	53
Finland	62	53
Norway	29	52
Poland	103	52
Portugal	56	50
Taiwan	113	50
Malaysia	82	48
Iran	79	46
Austria	50	43
Ireland	31	43
Brazil	102	42
Greece	42	30
Saudi Arabia	31	30
Russia	69	28
Turkey	74	28
Egypt	37	27
Pakistan	37	27
New Zealand	30	25
Vietnam	15	25
Mexico	32	23
Israel	48	22
Hungary	14	18
Romania	25	16
Algeria	8	15
South Africa	29	15
United Arab Emirate	15	15
Indonesia	90	14
Qatar	7	14
Colombia	14	12
Ecuador	26	11
Czechia	20	10
Serbia	14	10
Chile	14	9

^9^, and

**Table 10:** Coupling structural network in machine learning

Source	Documents	Total link strength
Lecture notes in computer science	467	8618
IEEE access	94	3500
PLoS one	129	3140
BMC bioinformatics	42	1893
IEEE transactions on image processing	33	1769
IEEE Transactions on pattern analysis and machine learning	29	1735
Sensors	56	1622
Advances in intelligent systems and computing	137	1621
Studies in computational intelligence	41	1214
Proceedings of Spie	101	1189
Multimedia tools and application	33	1078
Bmc genomics	27	1032
Neural computing and application	33	1078
Biorxiv	44	950
IEEE journal of biomedical and health informatics	22	934
IEEE transactions on neural networks and learning	30	863
IEEE transactions on medical imaging	9	729
Neural Networks	20	768
IEEE transactions on geoscience and remote sensing	18	1682
IEEE transactions on multimedia	14	578
Neural computation	8	563
IEEE transaction on circuits and systems for video	8	557
Springer handbooks of computational statistics	11	551
Knowledge and information systems	21	548
Remote sensing	11	539
Journal of biomedical informatics	11	539
Neuroimage	23	539
Artificial intelligence in medicine	12	516
Computer methods and programs in biomedicine	13	513
IEEE transactions on signal processing	22	507
Computational and mathematical methods in medicine	11	498
IEEE transactions on signal processing	21	495
Communication in computer and information science	63	481
Scientific reports	11	448
Bioinformatics	18	429
Machine learning	12	412
Plos computational biology	15	409
Medical image analysis	7	404
International Journal of computer vision	6	402
Artificial intelligence review	11	392
International journal of machine learning and cybersecurity	14	379
IEEE transactions on visualization and computer graphics	6	375
Springerbriefs in computer science	5	375
Algorithms in computational molecular biology	10	367
Entropy	12	359
Bmc system biology	14	352
Computers in biology and medicine	12	343
Neuroscience	5	343
Soft computing	13	341
The scientific world journal	18	306

^10^.
